# Frequency and Complications of Ileostomy

**DOI:** 10.7759/cureus.11249

**Published:** 2020-10-29

**Authors:** Ayesha Mehboob, Sughra Perveen, Mazhar Iqbal, Kulsoom Moula Bux, Abdul Waheed

**Affiliations:** 1 General Surgery, Jinnah Postgraduate Medical Centre, Karachi, PAK; 2 General Surgery, Jinnah Post Graduate Medical Centre, Karachi, PAK

**Keywords:** keywords: ileostomy, ileostomy hernia, ileostomy prolapse

## Abstract

Introduction

Ileostomies are life-saving procedures used for temporary fecal diversion in complicated cases of ileal perforation. However, an ileostomy is associated with several complications. The goal of this study was to determine the frequency and complications of ileostomy in the Jinnah Postgraduate Medical Centre, Karachi, Pakistan.

Methodology

We conducted a longitudinal observational study from July 2019 to July 2020. All patients older than age 12 receiving an ileostomy were included. Patient examinations were conducted on the first postoperative day and were assessed for hemorrhage and necrosis. Examinations were conducted on the seventh postoperative day to assess stoma retraction, stenosis, prolapse, and high-output fistula. Patients were monitored via follow-up in the outpatient clinic every 15 days to record any complications for three months until the reversal was performed. All data were analyzed using IBM SPSS Statistics for Windows, Version 25.0. (Armonk, NY: IBM Corp.).

Result

A total of 84 patients who received ileostomies were included in the study. Most patients were male (n=62; 73.8%), and 22 were female (26.19%). Of the 84 patients in our study, 34 (40.48%) had tuberculous intestine, 23 (27.38%) had typhoid ileal perforation, 23 (27.38%) were trauma patients, and four patients (4.7%) had gangrenous appendicular perforation. 23 patients (27.3%) were discharged with no complications, and 61 patients (72.69%) developed complications. The most common complication was skin excoriation (19.4%), followed by wound infection (13%), nonfunctioning stoma (11.9%), prolapse and stenosis (6%), retraction (4.7%), high-output fistula (3.5%), parastomal hernia and necrosis (2.3% each), and hemorrhage (1.1%).

Conclusion

Ileostomy is a common and lifesaving surgical diverting procedure. It is still common in our clinic due to late presentation by patients who need the procedure. Complications are common but manageable. Therefore, it is essential to recognize these complications and manage them early to reduce the morbidity of the patients.

## Introduction

An ileostomy can decrease morbidity and mortality associated with anastomotic leakage in the colonic and small gut anastomosis. Ileostomies can prevent morbidity in septicemic patients of ileal perforation due to typhoid fever, tuberculosis, trauma, or ruptured appendix; however, complications (e.g., stomal obstruction, skin excoriation, dehydration due to high ileostomy output causing fluid and electrolyte loss) can occur in up to 16.9% of cases within 60 days [1,2]. A diverting ileostomy created during surgical treatment is associated with morbidity but is also a life-saving procedure when indicated. A combination of fluid resuscitation or antimotility therapy and adjusted dietary regimen is indicated in patients with ileostomy complications [3]. The typical output from an ileostomy should not exceed 1500 ml per day [3].

Ileostomy complications cause laceration from clotting, mucocutaneous suppuration, stoma separation from the skin, and peritonitis. Stoma necrosis can occur due to strangulation and low blood flow from the surgical procedure. Stoma prolapse occurs when the stoma is displaced and proximal bowel slides through the side of the stoma orifice [4]. Stoma retraction occurs when the stoma is reduced approximately 5 cm below the skin surface; this late complication occurs after weight gain. Stoma retraction may occur only in adhesion, sepsis, radiation of intestine [5], and certain surgical technique [6]. Bowel obstruction is a common complication and requires surgical intervention [7]. There is a high rate of complication in loop ileostomies, but they are not life-threatening. An ileostomy can prevent life-threatening complications due to anastomotic leakage.

Most ileostomy complications are treated with surgical intervention. Therefore, this study was conducted to determine the frequency of complications in our tertiary care center, where many cases of late presenting acute peritonitis are treated via ileostomy.

## Materials and methods

We conducted this longitudinal observational study in Ward Three of the Jinnah Postgraduate Medical Centre from July 2019 to July 2020. Patients older than age 12 receiving ileostomy were included in the study. The study included patients of peritonitis due to typhoid perforation, tuberculosis intestine, right hemicolectomy due to gangrenous appendix, and colonic perforation or obstruction. Excluded from the study were any patients with metastatic colorectal tumors. Indication of ileostomy was septicemia due to late presentation (>48 hours) or covering ileostomy in colonic resection and anastomosis.

Ileostomies were end ileostomy in right hemicolectomy and loop ileostomies in cases of ileum perforation or colonic resection anastomosis. Patient examinations were conducted on the first postoperative day, and we assessed for hemorrhage and necrosis, defined as a dusky, dark appearance of the ileostomy that did not subside even after the edema settled. Examinations were conducted on the seventh postoperative day to assess for stoma retraction (defined as the stoma sunken 5 cm below the skin), stenosis, prolapse (defined as the bowel protruding >5 cm through the stomal opening), and high-output fistula (defined as >1500 ml/day with elevated patient pulse rate). Stoma were considered nonfunctioning if the stoma did not work for more than three days from the date of the procedure. Patient’s electrolyte levels were maintained during their hospital stay. Patients were monitored via follow-up in the outpatient clinic every 15 days to record any complications for three months until the reversal was performed.

Reversal of the ileostomies for cases of typhoid perforation, trauma, and right hemicolectomy due to a gangrenous appendix was performed in three months to allow time for these malnourished patients to become nourished appropriately prior to the procedure. Reversals of the ileostomies due to tuberculous were performed after six months. All patients who developed complications of prolapsed or retraction underwent early ileostomy closure, had a smooth recovery and did not encounter further complications. Patients who developed retraction or prolapse within the first week had their reversal performed during the same admission. All data were analyzed using IBM SPSS Statistics for Windows, Version 25.0 (Armonk, NY: IBM Corp).

## Results

**Figure 1:**
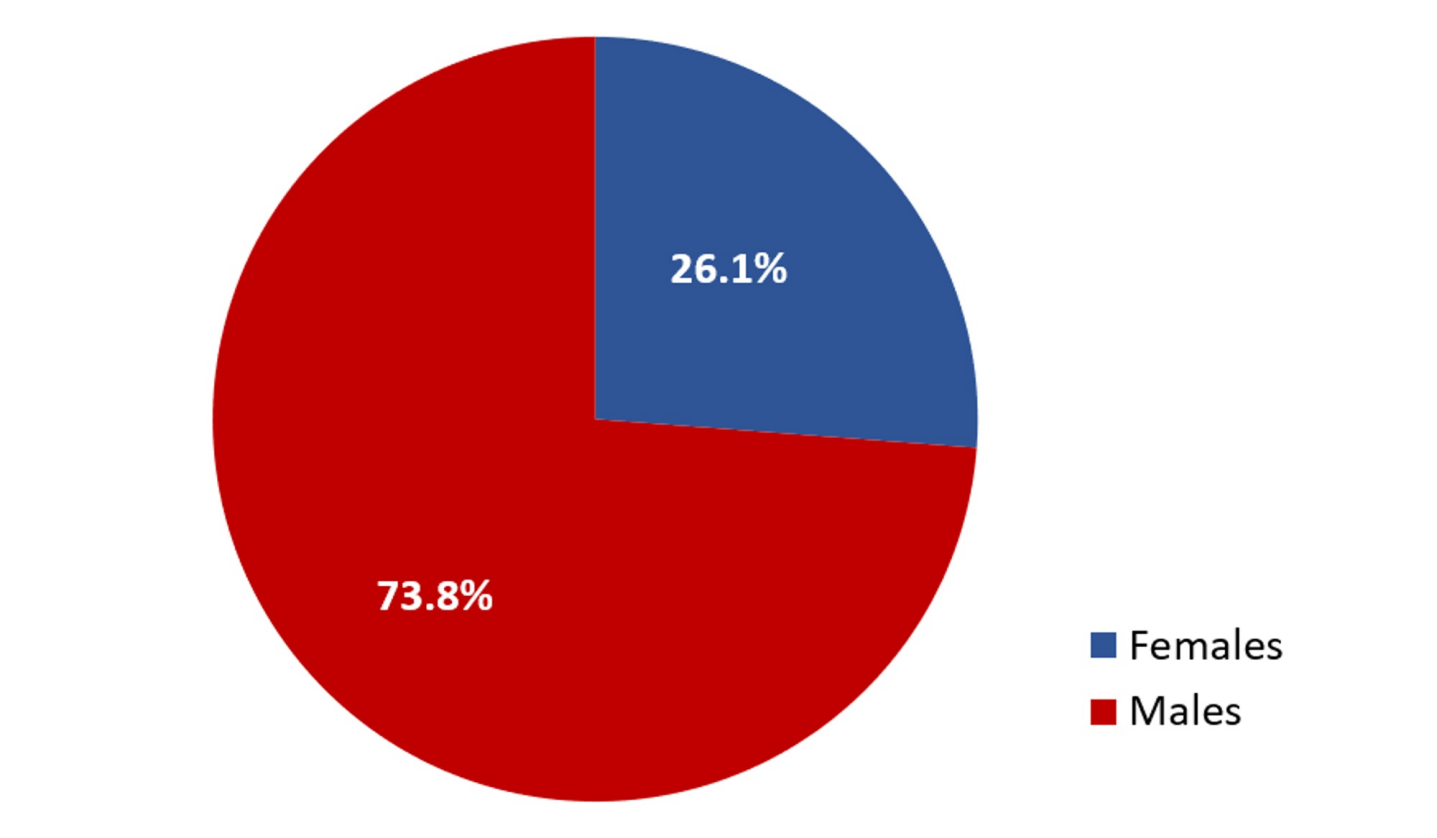
Male to female ratio Female patients (n=22); male patients (n=62)

A total of 84 patients received ileostomies following exploratory laparotomy due to acute peritonitis and were included in our study. Figure 1 presents the male to female ratio. Seven patients (8.33%) were age 13 to 20 years, 35 (41.66%) were age 21 to 30 years, 31 (36.9%) were age 31 to 40 years, and eight patients (9.5%) were age 41 to 50 years. Of the 84 patients in our study, 34 (40.48%) had tuberculous intestine, 23 (27.38%) had typhoid ileal perforation, 23 (27.38%) were trauma patients, and four patients (4.7%) had gangrenous appendicular perforation. Twenty-three patients (27.3%) were discharged with no complications, and 61 patients (72.69%) developed complications (Table 1). In all patients with complications, Ileostomy revisions were performed in all patients who developed complications.

**Table 1:** Occurrence of ileostomy complications (n=84)

Complication	Patients (n)	Percentage (%)
Uneventful	23	27.3%
Skin excoriation	16	19.4%
Wound Infection	11	13.09%
Nonfunctioning stoma	10	11.9%
Prolapse	6	7.1%
Stenosis	6	7.1%
Retraction	4	4.7%
High output fistula	3	3.5%
Parastomal hernia	2	2.3%
Necrosis	2	2.3%
Hemorrhage	1	1.1%

## Discussion

An ileostomy is a life-saving procedure to divert the fecal stream from the colon to protect downstream anastomosis, and the procedure consists of bringing the lumen of the ileum through the abdominal wall via a surgical opening [8]. Ileostomies can be temporary or permanent and are indicated for a wide array of gastrointestinal disorders. Indications include typhoid ileal perforation, tuberculous intestines, and bowel obstruction/stool evacuation for situations in which the colon has been removed, such as in colorectal cancers, ulcerative colitis, Crohn’s disease, and familial adenomatous polyposis.

Healthcare providers should retain a basic skill and knowledge on creating stomas, including their management and complications, as crisis managers for ostomates [9]. Ileostomies can dramatically improve the quality of life for a patient.

Complications related to stoma are both frustrating and stressful for both surgeons and patients [10]. Stoma complications have significant morbidity and include necrosis, prolapse, retraction, skin excoriation, parastomal hernia, high-output fistula, stenosis, hemorrhage, nonfunctioning stomas, and wound infections [11].

An international study of 279 ileostomy patients reported an overall complication rate of 83%, similar to our 72.6% rate [12]. Both this study and the international study reported skin-related complications as most common: 19.4% of our study participants had skin excoriation, and 47% of patients in the international study had peristomal dermatitis. Our complications were comparatively lower because operations were performed earlier and with better techniques. In an international study conducted by Bhama et al., participants had a lower rate of complications (20%) compared to our study (72%) [13]. The difference may be due to Bhama et al.’s smaller sample size and better sterile techniques. However, our patients presented late due to fear of the operation and low education levels, where the need to seek medical help was not taken seriously and, therefore, delayed, which increased the septic load and more complications than those in the Bhama study.

Skin excoriation and ulceration are common and occur when the skin is exposed to ileostomy contents. This causes enzymatic digestion of skin proteins, damaging the skin. Skin excoriation was the most common complication in our study (19.4%) and was noted in 25% of ileostomy patients in a study be Ambe et al. [14]. We found that applying a zinc paste topical treatment seemed to help the patients recover.

Nonfunctioning stoma occurred in 11.9% of patients in our study, which we addressed with immediate revision with lateral fixation. Maatouk et al. used urgent conservative surgical management with manual reduction to treat patients with a nonfunctioning stoma in their study [15].

Prolapsed stoma occurred in 7.1% of patients in our study, which was a higher prevalence than those in the Li et al. study, which reported prolapse in only 4.2% of patients [16]. Prolapse can be prevented by internal fixation done by a skilled surgeon.

A stoma that has retracted causes significant difficulties. The contents that spill over the skin causes excoriation, pain, and infections. Furthermore, it can cause abscesses and collections in the abdomen via the sunken area, leading to peritonitis [17]. The retraction rate in our study was 4.7% and can be prevented by adequately suturing the bowel with the skin.

High-output fistula (or high-output syndrome) is a dreadful complication of ileostomy that usually occurs in the first 15 days after the operation and results in fluid loss along with electrolyte and protein loss. Our high-output fistula complication rate was 3.5%, which was much lower than that reported by Takeda et al. (23.8%) [18] and Justiniano et al. (26%) [19]; this is because we made the ileostomies close to the terminal ileum which produces adequate output. Because of this, we had a low incidence of high-output fistulas in our patients.

A peristomal hernia is a complication comprising a bulge around the site of the stoma, and coughing can exacerbate this complication. Our rate of peristomal hernia was only 2.3%, which was lower than the rate reported by Maatouk et al. (10.1%) [15]. A peristomal hernia can be prevented by minimizing the incision.

Necrosis is a significant early complication that results from an inadequate stomal blood supply. The stoma appears dusky and swollen, thereby narrowing the stoma and interfering with the blood outflow. The blood flow usually returns after the edema resolves. However, if the blood supply is devascularized during ligation in surgery, immediate revision is required. The rate of necrosis in our study was 2.3%, which was lower than the rate reported by Krishnamurty et al. (13%) [20].

Our study was limited in that emergencies delayed the surgical operations, and several patients were lost to follow-up (and excluded from the study).

## Conclusions

Ileostomy is a common and lifesaving surgical diverting procedure. It is still common in our clinic due to late presentation by patients. Complications are common but manageable. Therefore, it is essential to recognize these complications and timely manage them to reduce the morbidity of the patients.
